# Praseodymium‐Modulated NiFe Layered Double Hydroxide with Enhanced Oxygen Evolution Activity and Extended Durability

**DOI:** 10.1002/advs.78238

**Published:** 2026-10-12

**Authors:** Yuanming Gao, Xuezhen Wu, Lin Zhang, Haoxiang Zhang, Xueying Feng, Yu Bai, Wei Song, Yong Zheng, Jiong Liu, Jieyu Chen, Dong Yang, Wen Ma, Shengzhong (Frank) Liu

**Affiliations:** ^1^ Inner Mongolia Key Laboratory of New Materials and Surface Engineering School of Materials Science and Engineering Inner Mongolia University of Technology Hohhot China; ^2^ State Key Laboratory of Photoelectric Conversion and Utilization of Solar Energy Dalian Institute of Chemical Physics Chinese Academy of Sciences Dalian China

**Keywords:** electronic modulation, long‐term durability, NiFe layered double hydroxide, oxygen evolution reaction, praseodymium doping

## Abstract

The oxygen evolution reaction (OER) in alkaline water electrolysis suffers from sluggish kinetics and limited catalyst durability. Here, we present a praseodymium (Pr) doping strategy that simultaneously tailors the electronic structure and pore architecture of nickel–iron layered double hydroxide (NiFe‐LDH). Atomically dispersed Pr^3+^ expands the interlayer spacing from 0.76 to 0.81 nm and refines the mesoporous network, increasing Brunauer–Emmett–Teller (BET) surface area by 13.4% and decreasing the average BJH desorption pore size by 12.8%. Strong Pr─O covalency downshifts the Ni 3d band center and reinforces Ni─O hybridization, as corroborated by X‐ray absorption spectroscopy and density functional theory (DFT). These synergistic effects switch the OER rate‐determining step (RDS) from ^*^O → ^*^OOH (ΔG = 0.74 eV) to ^*^OH → ^*^O (ΔG = 0.28 eV). The optimized Ni_2_Fe_0.88_Pr_0.12_‐LDH delivers an overpotential of 407 mV at 500 mA cm^−2^ and sustains 2190 h in half‐cell tests. When integrated into an anion exchange membrane (AEM) electrolyzer, it operates continuously for over 1000 h at industrially relevant current densities, with a moderate voltage drift of 0.202 V (average degradation rate ∼0.20 mV h^−1^). This work establishes a dual electronic and pore engineering route for durable LDH‐based OER catalysts.

## Graphical abstract

Pr doping of NiFe‐LDH simultaneously tunes Ni 3d electronics and mesopore architecture. The optimized Ni_2_Fe_0.88_Pr_0.12_‐LDH switches the OER rate‐determining step, delivers 407 mV at 500 mA cm^−2^, and sustains 2190 h in half‐cell tests. An AEM electrolyzer with this anode operates over 1000 h at industrial current density, offering a durable rare‐earth‐doped LDH strategy for practical water electrolysis.

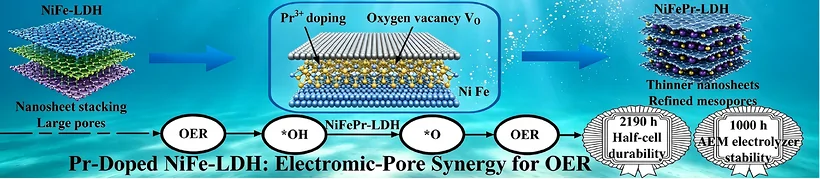

## 1 Introduction

Green hydrogen from water electrolysis is a key route toward carbon neutrality [1, 2, 3]. Among electrolysis technologies, alkaline water electrolysis (AWE) offers low capital cost but is hampered by the sluggish anodic oxygen evolution reaction (OER), a four‐electron/proton‐coupled process that requires high overpotentials, especially at current densities ≥500 mA cm^−2^ [4, 5, 6, 7]. Developing non‐noble‐metal OER catalysts with high intrinsic activity, fast mass transport, and long‐term durability remains a central challenge.

Layered double hydroxides (LDHs), especially NiFe‐LDH, are promising alkaline OER electrocatalysts owing to their tunable composition and abundant active sites [8]. Iron incorporation electronically activates Ni centers, facilitating formation of high‐valence NiOOH as the active phase [9]. Recent studies have explored rare‐earth (RE) doping, including Pr‐modified NiFe‐based (oxy)hydroxides, to modulate active‐site electronic structure via 4f orbital interactions [10, 11]. However, pristine NiFe‐LDH suffers from three intrinsic drawbacks: (i) severe nanosheet stacking obscures edge sites; (ii) adsorption energies of OER intermediates (^*^OH, ^*^O, ^*^OOH) deviate from the optimal range at high current densities; and (iii) prolonged electrolysis triggers metal leaching and structural reconstruction [12, 13]. Moreover, its disordered large‐pore structure impedes electrolyte penetration and mass transfer.

Heteroatom doping is an effective strategy to optimize LDH performance [12, 13, 14, 15]. RE ions, with unique 4f configurations, strong Lewis acidity, and high oxophilicity, can induce charge redistribution via RE–O–M bridges and modulate the d‐band center of adjacent metal sites [16, 17, 18, 19, 20]. In addition, the larger ionic radius of RE^3+^ (e.g., Pr^3+^: 0.099 nm vs. Ni^2+^: 0.069 nm, Fe^3+^: 0.065 nm) can expand interlayer spacing and refine pore structure. Nevertheless, a comprehensive understanding of the synergy between RE‐induced electronic reconstruction and pore refinement, and its correlation with high‐current OER performance, is still lacking.

Herein, we synthesize Pr‐doped NiFe‐LDH directly grown on nickel foam (NF) via a one‐step hydrothermal method [21, 22, 23]. We systematically investigate structural evolution, electronic modulation, pore optimization, and OER performance. This work integrates atomic‐scale electronic redistribution with nanoscale pore architecture refinement and bridges the gap to practical application by demonstrating an AEM electrolyzer exceeding 1000 h of operation. Key findings are threefold: (i) atomically dispersed Pr^3+^ substitutionally incorporated into the LDH layer expands interlayer spacing and constructs a 3D nanoflower architecture with refined mesopores, exposing more active sites; (ii) strong Pr─O interactions enrich electron density on Ni/Fe sites, lower formation energy of active NiOOH, and switch the OER RDS; (iii) the optimal Ni_2_Fe_0.88_Pr_0.12_‐LDH shows exceptional activity (η_500_ = 407 mV) and durability (2190 h half‐cell, >1000 h AEM electrolyzer). In situ Raman and DFT calculations confirm that Pr doping downshifts the Ni 3d band center, enhances Ni**─**O covalency, and reduces the RDS energy barrier from 0.74 eV to 0.28 eV. This work offers a practical RE modulation blueprint for robust OER catalysts.

## 2 Results and Discussion

### 2.1 Synthesis and Structural Characterization

Pr‐doped NiFe‐LDH catalysts were grown in situ on NF via a one‐step hydrothermal route (Figure 1a) [21, 22, 23]. A series of Ni_2_Fe_1‐x_Pr_x_‐LDH samples with Pr contents of 1–5 mol% relative to total metals (x = 0.03, 0.06, 0.09, 0.12, 0.15) were prepared. X‐ray diffraction (XRD) was used to analyze the crystal structures (Figure 1f, Figures S1k,l). The NF‐supported catalysts and exfoliated powder exhibit characteristic LDH peaks (PDF#00‐051‐0463), while metallic Ni substrate peaks (PDF#00‐004‐0850) are also observed for the NF‐supported samples, confirming the phase‐pure LDH structure [24, 25]. The (003) diffraction peak progressively shifts to lower angles with increasing Pr content up to x = 0.12, confirming that Pr**^3+^** enlarges the LDH interlayer spacing from 0.76 to 0.81 nm. At x = 0.15, new diffraction peaks assigned to Pr_2_O_3_ (PDF#97‐060‐0743) emerge, indicating segregation of excess Pr species [26]. Inductively coupled plasma optical emission spectroscopy (ICP‐OES) (Table S1) shows that actual Ni/Fe/Pr molar ratios in the exfoliated catalyst layer agree with the designed compositions within <3% deviation; the catalyst loading on NF was estimated to be ∼1.77 mg cm^−2^. Because whole‐electrode ICP‐OES is dominated by the NF substrate, composition verification was performed on exfoliated catalyst powder, whereas whole‐electrode ICP‐OES was used for leaching analysis. Raman spectra (Figure 1g) show two characteristic peaks at 456 cm^−1^ (E*_g_* Ni**─**O bending) and 532 cm^−1^ (A*_g_* hydrotalcite stretching). No Pr_2_O_3_ signals are detected, further proving the atomic‐level dispersion of Pr in LDH lattice.

**FIGURE 1:**
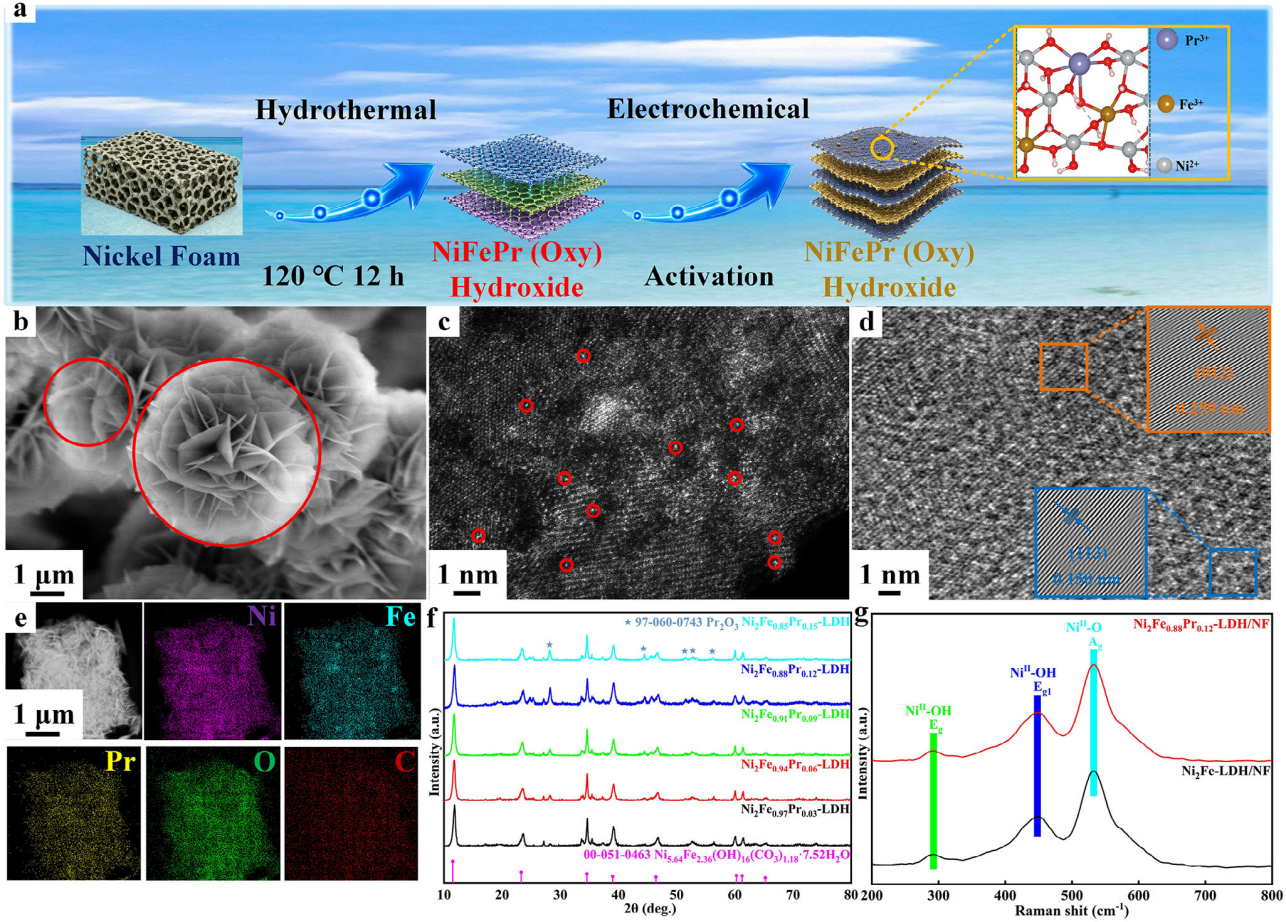
Morphology, crystal structure and elemental distribution of Pr‐doped NiFe‐LDH catalysts. (a) Schematic diagram of the one‐step hydrothermal synthesis and electrocatalytic evaluation process. (b) SEM image. (c) Aberration‐corrected HAADF‐STEM image. (d) HRTEM image. (e) STEM‐EDS elemental mapping. (f) XRD patterns. (g) Raman spectra.

Morphology and microstructures were characterized by **scanning electron microscopy (SEM)**, **transmission electron microscopy (TEM)**, aberration‐corrected high‐angle annular dark‐field scanning transmission electron microscopy (**HAADF‐STEM**), high‐resolution TEM (**HRTEM**), selected‐area electron diffraction (**SAED**), and STEM‐energy‐dispersive X‐ray spectroscopy (**STEM‐**EDS). SEM images (Figure 1b, Figure S1a–c) reveal a nanoflower‐like morphology similar to that of conventional NiFe‐LDH; Pr doping mainly refines the mesoporous architecture within the nanoflower morphology and drives ultrathin LDH nanosheets to assemble uniformly on the NF skeleton. This architecture prevents nanosheet stacking and exposes more active surfaces. BET and BJH analyses (Figure S2, Table S2) quantitatively confirm pore refinement. TEM images (Figure S1d–f) confirm the intact sheet‐like morphology of the exfoliated LDH. Aberration‐corrected HAADF‐STEM (Figure 1c) resolves isolated bright spots with an average size of 0.21 ± 0.03 nm, based on >60 randomly selected spots (Figures S1h–j). This size is consistent with substitutionally occupying Ni^2+^/Fe^3+^ sites by Pr^3+^ rather than formation of larger PrO_x_ clusters. HRTEM (Figure 1d) shows well‐resolved lattice fringes with d‐spacings of 0.259 and 0.150 nm, indexed to the (012) and the (113) planes of LDH. The matching SAED pattern (Figure S1g) verifies high crystallinity and lattice expansion after Pr doping. STEM‐EDS elemental mapping (Figure 1e) and HAADF‐STEM‐EDS elemental mapping (Figures S1m,n) show homogeneous distribution of Ni, Fe, Pr, O, C, and N across the nanosheet, supporting uniform Pr doping and good structural integrity.

### 2.2 Electronic Structure and Local Coordination Environment

X‐ray photoelectron spectroscopy (XPS) and synchrotron X‐ray absorption fine structure (XAFS) were used to probe electronic and coordination changes induced by Pr doping [27, 28]. The Ni 2p and Fe 2p spectra were fitted using multiplet envelopes and Shirley background subtraction, following established procedures for first‐row transition‐metal oxides/hydroxides [29, 30]. In the Ni 2p and Fe 2p spectra (Figure 2a–c), both **peaks shift continuously to lower binding energy with increased Pr loading, reaching a total shift of about −0.3 eV for the optimal sample**, which indicates increased electron density on Ni and Fe sites, consistent with the Ni^2^^+^/Ni^3^^+^ and Fe^2^^+^/Fe^3^^+^ binding energies reported for NiFe‐LDH systems [31]. The Pr 3d spectra show two peaks at 934.6 eV (3d_5/2_) and 954.7 eV (3d_3/2_), assigned to Pr^3+^, with peak positions and spin‐orbit splitting consistent with reported Pr 3d fitting parameters for Pr^3+^‐based oxides [32]. No Pr**^4+^** signals are observed, and the peak intensity increases linearly with Pr content while peak positions remain unchanged, confirming a stable +3 valence for dispersed Pr. Supplementary XPS data (Figure S3a–c) support these results. The survey spectrum (Figure S3a) confirms the coexistence of Ni, Fe, Pr, O, and C. Deconvoluted O 1s spectra (Figure S3b) contain M─O, M─OH, defect oxygen, and carbonate species. The M─O peak shifts to lower binding energy and the defect oxygen intensity increases markedly, indicating strong Pr─O interactions and modulated surface oxygen configuration. C 1s spectra (Figure S3c) corresponding to interlayer carbonate groups show only slight shifts, indicating the LDH carbon framework remains intact.

**FIGURE 2:**
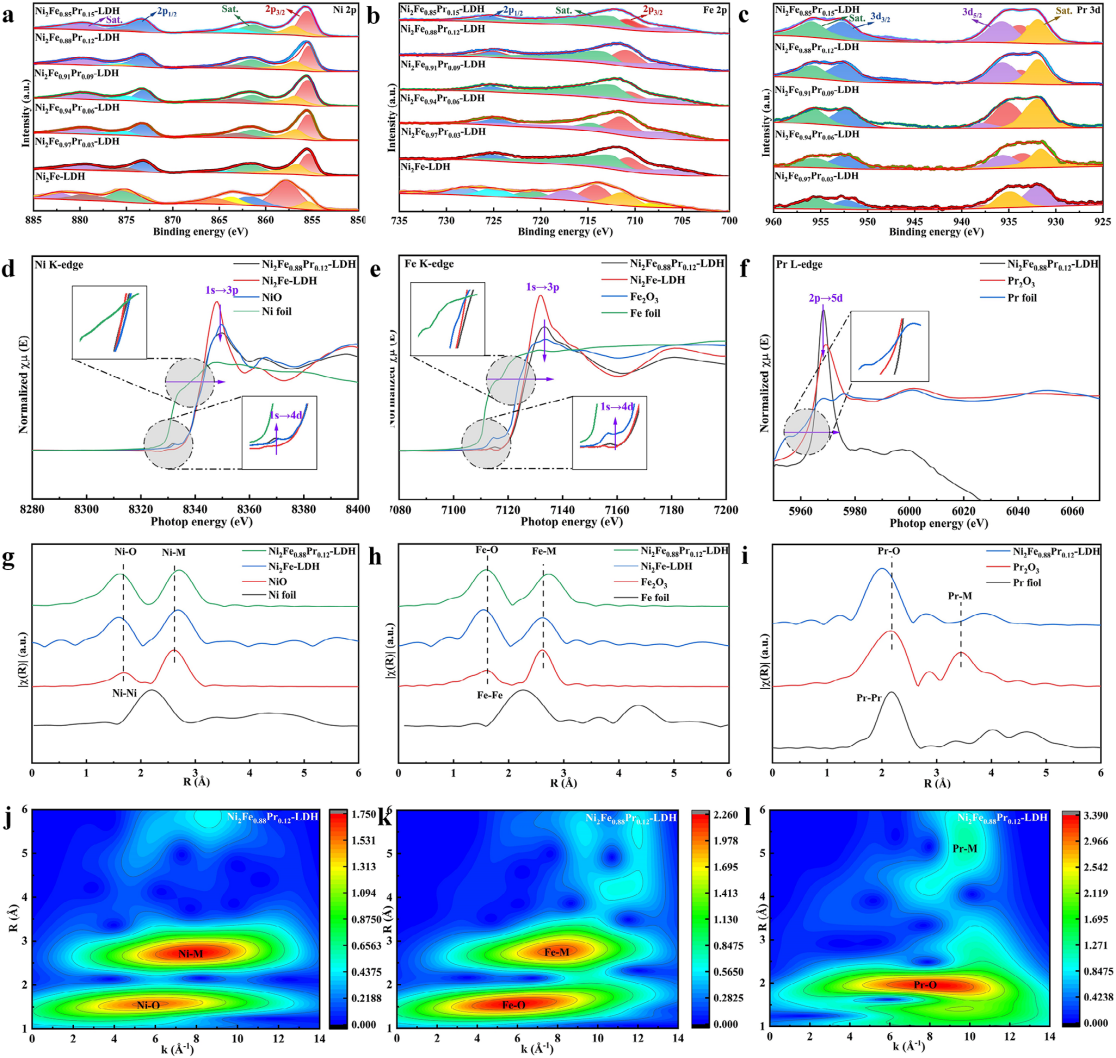
Electronic structure and local coordination environment of Pr‐doped NiFe‐LDH catalysts. (a–c) High‐resolution XPS spectra of Ni 2p, Fe 2p and Pr 3d. (d, e) Ni K‐edge and Fe K‐edge XANES spectra of Ni_2_Fe_0.88_Pr_0.12_‐LDH, undoped Ni_2_Fe‐LDH, and the reference oxides (NiO, Fe_2_O_3_). (f) Pr L‐edge XANES spectrum. (g–i) R‐space EXAFS spectra of Ni K‐edge, Fe K‐edge and Pr L‐edge. (j–l) WT‐EXAFS contour plots of Ni K‐edge, Fe K‐edge and Pr L‐edge.

XAFS was further revealed to probe bulk electronic states and local coordination. The Ni K‐edge and Fe K‐edge XANES of Ni_2_Fe_0.88_Pr_0.12_‐LDH (Figure 2d,e) show clear negative shifts relative to NiO/Fe_2_O_3_ references and undoped Ni_2_Fe‐LDH, revealing reduced average oxidation states of Ni and Fe after Pr doping [33, 34]. Pr L_3_‐edge XANES (Figure 2f) agrees well with the Pr_2_O_3_ standard, further verifying Pr^3+^. The Pr L_3_‐edge was measured in fluorescence mode; because self‐absorption and sample‐thickness effects can cause the post‐edge normalized intensity to deviate from unity, the valence assignment relies on edge position and EXAFS analysis rather than the absolute normalized intensity. In R‐space EXAFS (Figure 2g–i), **the Ni─O bond length elongates from 1.590 Å (pristine LDH) to 1.618 Å, and the Fe**─**O bond length reaches 1.603 Å. These fitting protocols follow established practices** [35, 36, 37]. The Pr L_3_‐edge EXAFS (Figure 2i, Figure S3i) shows a dominant Pr**─**O peak at ∼1.998 Å (CN ≈ 6.2) and a weak Pr**─**M peak at ∼3.847 Å. The fitted Pr**─**O CN matches the octahedral coordination environment of the LDH layer rather than the low‐coordination environment typical of surface adatoms. We therefore conclude that Pr is atomically dispersed as a substitutional dopant within the LDH lattice, not as a surface single‐atom decoration. No Pr**─**Pr or metal–metal scattering signals are detected, ruling out metal clusters and confirming atomic dispersion of the metal species. Complete EXAFS fitting parameters, including k‐range, R‐range, N, R, σ**^2^**, ΔE_0_, and R‐factor, are summarized in Table S3, together with NiO, Fe_2_O_3_, Ni foil, and Fe foil references. The asymmetric Ni**─**O/Fe**─**O peaks around 1.5–2.0 Å in R‐space (Figure 2g,h) were further examined. Extending the k‐range to ∼12 Å^−1^ (Figures S2d–f) did not remove the asymmetry, indicating that it is not an artifact of k‐range truncation. Instead, the asymmetry is primarily attributed to substitutional incorporation of the larger Pr^3+^ ion, which induces local lattice distortion and creates multiple Ni/Fe**─**O coordination environments with slightly varying bond lengths through Pr**─**O**─**M bridges. Overlapping coordination shells produce the asymmetric envelope. This interpretation is strongly supported by WT‐EXAFS (Figure 2j–l), which shows an irregular intensity distribution in the 8–12 k Å^−1^ range corresponding to R‐space of 2–3 Å—a feature commonly observed in heteroatom‐doped layered hydroxides, where dopant‐induced local structural heterogeneity modulates the adsorption energetics of catalytic intermediates [38]. The refined local coordination environment correlates with the reduced ^*^OH → ^*^O step in our DFT calculations (Figure 4a). Wavelet transform EXAFS (**WT‐EXAFS**) contour plots (Figure 2j–l, Figure S4a,d) resolve local coordination in k and R space. All signals are concentrated in the M**─**O and M**─**M regions without high‐R metal agglomeration, in good agreement with the EXAFS results. The larger ionic radius of Pr^3+^ thus induces LDH lattice expansion. Strong Pr**─**O covalent bonds polarize oxygen electron clouds and drive electron transfer to adjacent Ni**─**O/Fe**─**O bridges. **Such electron enrichment on Ni sites greatly reduces the formation energy of active NiOOH and optimizes the adsorption of OER intermediates** [33, 34]. A detailed EXAFS comparison with undoped Ni_2_Fe‐LDH control is provided in Figure S4, further confirming the lattice expansion and coordination‐environment heterogeneity induced by Pr incorporation.

### 2.3 Electrocatalytic OER Performance

The OER performance was evaluated in 1.0 m KOH electrolyte [39, 40]. Hydrothermal conditions were first optimized. **Electrochemical tests (Figure** S5 **) confirm that the catalyst synthesized at 120°C for 12 h delivers the best activity**. Lower temperature (100°C) leads to incomplete crystallization, while higher temperatures (140–160°C) cause excessive crystal growth, aggregation, and inactive NiO impurities. The 120°C/12 h sample also has the smallest Tafel slope (63.27 mV dec^−1^), indicating the fastest OER kinetics among the hydrothermal series. Ni/Fe ratio screening (Figure S6a–c, f, i) identifies a 2:1 ratio as optimal, with overpotential of **225 mV at 10 mA cm** ^−^ **^2^ and 379 mV at 100 mA cm** ^−^ **^2^, a Tafel slope of 52.3 mV dec** ^−1^, the lowest charge‐transfer resistance, and the largest double‐layer capacitance (**C_dl_**) among Ni_x_Fe‐LDH/NF. This composition is therefore selected as the baseline for subsequent RE doping.

La, Ce, Pr, and Nd were screened at identical loading by linear sweep voltammetry (**LSV**), overpotential statistics, Tafel plots, electrochemical impedance spectroscopy (**EIS**), and **C_dl_** (Figure 3a–c, Figure S6d,g). Pr doping gave the lowest overpotential, smallest charge‐transfer resistance, and largest C_dl_, identifying Pr as the optimal dopant [41, 42, 43]. Graded Pr contents in Ni_x_Fe_1‐x_Pr_x_‐LDH/NF were then tested (Figure 3d–f, Figure S6e,h, Table S4). The optimal Ni_2_Fe_0.88_Pr_0.12_‐LDH requires only 198 mV at 10 mA cm^−^ **^2^** and 407 mV at 500 mA cm^−^ **^2^**, whereas pristine Ni_2_Fe‐LDH/NF requires 939 mV at 500 mA cm**^−2^**. It also shows a small Tafel slope of 53.0 mV dec^−1^. Its turnover frequency (TOF) at η = 0.2 V reaches 0.0207 s^−1^ (Figure 3g), 3–5 times higher than other Pr‐doped samples, indicating superior intrinsic activity. ECSA‐normalized LSV curves (Figure S6k) confirm that the enhancement originates from Pr doping rather than surface‐area effects [44]. High‐current LSV (Figure S6l) shows that Ni_2_Fe_0.88_Pr_0.12_‐LDH/NF reaches 500 mA cm^−2^ at 1.637 V vs. RHE (η = 407 mV), compared with 2.169 V vs. RHE for undoped Ni_2_Fe‐LDH/NF.

**FIGURE 3:**
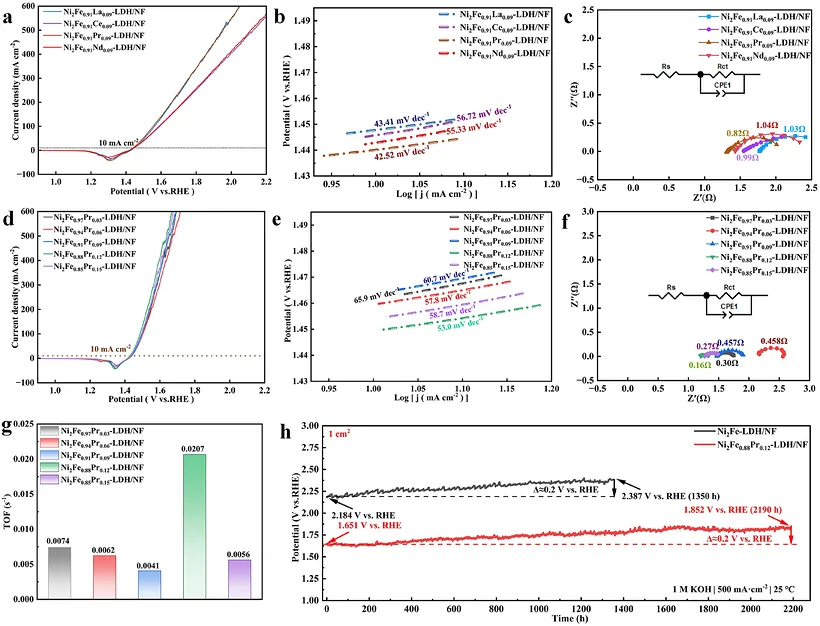
Electrochemical OER performance of Pr‐doped NiFe‐LDH catalysts. (a–c) iR‐corrected LSV curves recorded at 5 mV s^−1^ in 1.0 m KOH; corresponding Tafel slopes and EIS Nyquist plots with an equivalent circuit model of Ni_2_Fe_0.91_RE_0.09_‐LDH (RE = La, Ce, Pr, Nd). (d–g) iR‐corrected LSV curves recorded at 5 mV s^−1^ in 1.0 m KOH, Corresponding Tafel slopes, EIS Nyquist plots with equivalent circuit model, and TOF values of Ni_2_Fe_1‐x_Pr_x_‐LDH with various Pr contents. (h) Long‐term chronopotentiometric durability test of Ni_2_Fe‐LDH and Ni_2_Fe_0.88_Pr_0.12_‐LDH at 500 mA cm^−2^ in 1.0 M KOH.

N_2_ adsorption‐desorption (Figure S2b–e, Table S2) reveals an optimized pore structure after Pr doping: BET surface area increases by 13.4%, BJH average pore size decreases by 12.8%, cumulative pore volume rises by 4.8%, and cumulative surface area increases by 20.1%. This refined mesoporous architecture is beneficial for electrolyte diffusion and gas release, as generally recognized in mesoporous electrocatalysts. Bode plots of EIS (Figure S5j) show a slightly reduced phase‐angle plateau in the medium‐frequency region for the Pr‐doped sample, implying accelerated ion transport and consistent with established protocols for LDH‐based electrocatalysts [45]. Because direct in situ evidence of mass‐transport dynamics is still under investigation, this is presented as a structural advantage rather than a rigorously proven dynamic effect.

Long‐term durability at 500 mA cm^−2^ was assessed (Figure 3h, Figure S6l,Table S4). The overpotential of pristine Ni**_2_** Fe‐LDH rises by 200 mV after 1350 h, whereas Ni_2_Fe_0.88_Pr_0.12_‐LDH requires 2190 h to reach the same drift, a ∼1.6‐fold improvement in service life. Post‐mortem characterizations (TEM, SAED, XRD, XPS, and elemental mapping; Figures S7 and S8) were performed after 200 h as an intermediate chemical stability assessment. They confirm preserved nanosheet morphology, crystalline LDH phase, homogeneous elemental distribution, and stable Ni^2+^/Fe^3+^/Pr^3+^ states. ICP‐OES of recovered anodes after 200 h half‐cell OER (Table S5) provides quantitative evidence. For Ni_2_Fe_0.88_Pr_0.12_‐LDH, Ni, Fe, and Pr contents remain at 96.9845, 1.4395, and 0.0785 wt.%, respectively, close to nominal composition and indicate negligible leaching of host metals and Pr. Pristine Ni_2_Fe‐LDH retains Ni and Fe at 97.1872 and 0.8234 wt.%. The preserved Ni/Fe/Pr ratio confirms that Pr is incorporated into the LDH lattice rather than merely surface‐adsorbed. At a catalyst loading of ∼1.77 mg cm^−2^, absolute Fe and Pr loss after 200 h are ∼0.33 and ∼0.075 µg cm^−2^, respectively. These values are small relative to total catalyst loading, supporting the superior long‐term durability [46].

### 2.4 Mechanistic Insights from In Situ Raman and DFT

In situ Raman spectroscopy and DFT calculations were combined to reveal the origin of enhanced OER performance [47, 48]. In situ Raman spectra (Figure 4f) were collected from open circuit to 1.57 V vs. RHE. With increasing potential, the characteristic Ni*^II^*–OH (459 cm^−1^) and Ni*^II^*–O (536 cm^−1^) bands gradually shift to higher wavenumbers. At 1.52 V vs. RHE, they fully transform into NiOOH signals, directly confirming that Ni sites participate in the OER‐active phase. This does not exclude synergistic Fe participation, since Fe is known to electronically modulate Ni centers through Fe**─**O**─**Ni interactions, consistent with our DFT results.

**FIGURE 4:**
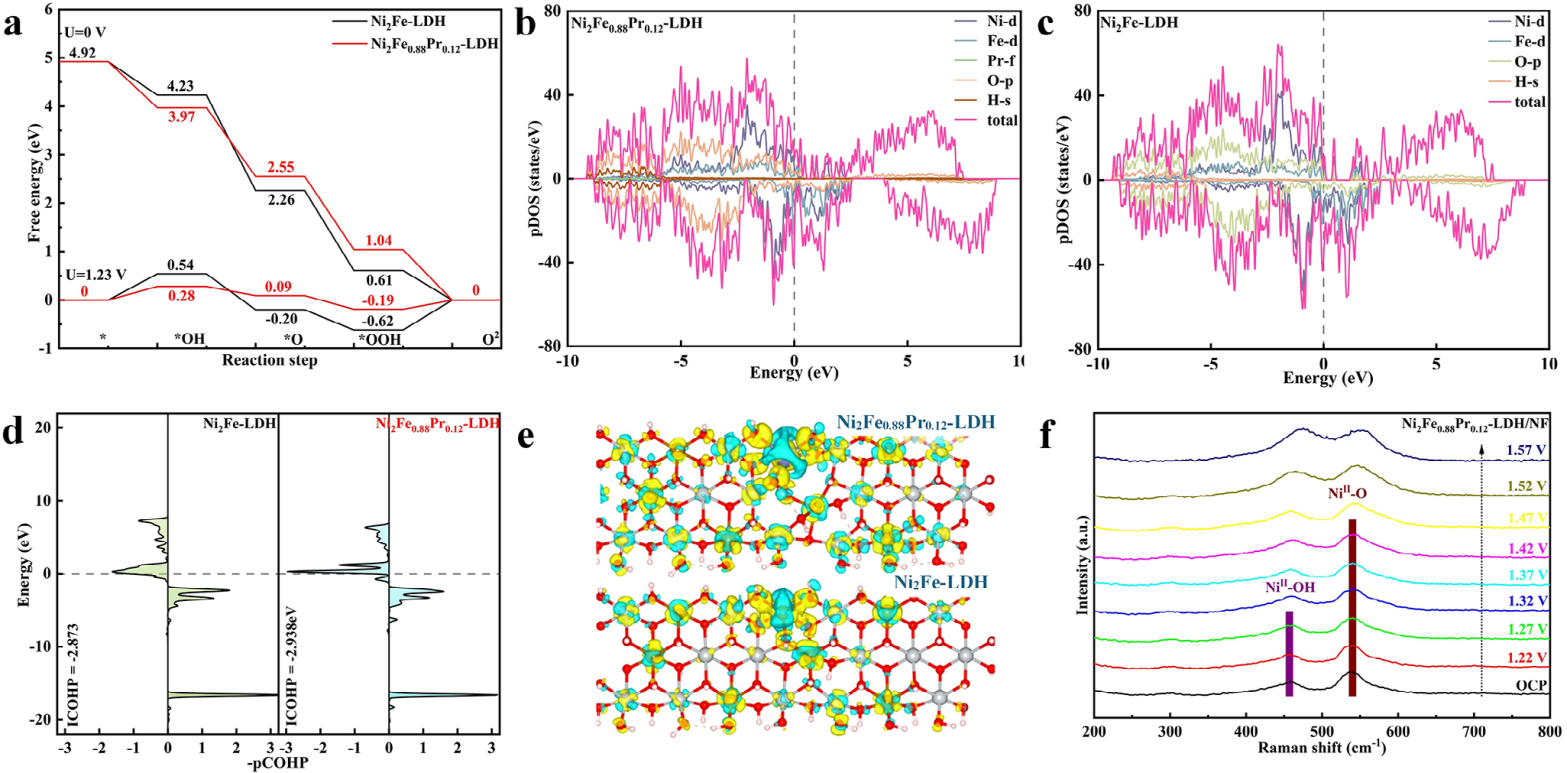
Catalytic mechanism investigation of Ni_2_Fe_0.88_Pr_0.12_‐LDH. (a) Gibbs free energy diagrams for the OER pathway at 0 and 1.23 V vs. RHE. (b, c) PDOS of Ni_2_Fe‐LDH and Ni_2_Fe_0.88_Pr_0.12_‐LDH. (d) COHP analysis of Ni**─**O bonds. (e) Differential charge density maps of Ni_2_Fe‐LDH and Ni_2_Fe_0.88_Pr_0.12_‐LDH. (f) In situ Raman spectra of Ni_2_Fe_0.88_Pr_0.12_‐LDH recorded at potentials from open circuit to 1.57 V vs. RHE.

DFT calculations further clarify the mechanism. **Gibbs free energy diagrams (**Figure 4a) show that RDS on pristine Ni_2_Fe‐LDH is ^*^O → ^*^OOH, with a high energy barrier of 0.74 eV. After Pr doping, the RDS switches to ^*^OH → ^*^O and the barrier drops sharply to 0.28 eV, greatly accelerating overall kinetics. Projected density of states (**PDOS**; Figure 4b,c) shows that **Pr doping downshifts the Ni 3d band center from −1.75 to −1.82 eV and upshifts the O 2p band center from −2.22 to −2.18 eV,** enabling balanced adsorption of OER intermediates. Crystal orbital Hamilton population (**COHP)** analysis (Figure 4d) shows that the **integrated COHP (ICOHP) of Ni─O bonds changes from −2.873 eV (pristine LDH) to −2.938 eV (Pr‐doped LDH)**. A more negative ICOHP indicates stronger Ni**─**O covalency, which stabilizes the active interfaces. Optimized DFT models of OER intermediates (^*^OH, ^*^O, ^*^OOH) are shown in Figure S2a. **Differential charge density maps (**Figure 4e **, isosurface value = 0.005 e Å^−3^)** reveal pronounced electron transfer from Pr to Fe. Concurrently, XPS (Figure 2a,b) and XANES (Figure 2d,e) show increased electron density on Ni sites (negative binding energy shifts and reduced absorption‐edge energies). These observations are reconciled by a cascade charge‐transfer pathway: Pr → Fe → Ni via Pr**─**O**─**Fe**─**O**─**Ni bridges. Specifically, Pr^3+^, with high oxophilicity and unique 4f orbitals, donates electrons to the Fe**─**O network; the electron‐enriched Fe sites then redistribute excess charge to neighboring Ni sites through the shared O bridge. This cascade simultaneously enriches electron density on Ni (the primary OER active site) nd stabilizes Fe against excessive oxidation, is consistent with both the enhanced NiOOH formation kinetics and the retarded Fe dissolution observed by ICP‐OES after 200 h of electrolysis (Table S5). The electron enrichment on Ni is consistent with the Ni 3d band‐center downshift (Figure 4b), which optimizes intermediate adsorption and lowers the OER overpotential.

### 2.5 Aem Electrolyzer Performance

To evaluate practical industrial application potential, a **membrane electrode assembly (MEA)** with an active area of 4 cm^2^ was assembled using Ni_2_Fe_0.88_Pr_0.12_‐LDH as the anode and Ni‐Mo foam (NMF) as the cathode (Figure 5a) [49, 50, 51]. The AEM electrolyzer was tested in 1.0 M KOH at 60°C. The polarization curve recorded without iR correction (Figure 5b **) shows that the NMF||**Ni_2_Fe_0.88_Pr_0.12_ **‐LDH electrolyzer requires 1.739 V to reach 500 mA cm^−2^. The energy efficiency was calculated from the as‐measured, non‐iR‐corrected cell voltage: 70.73% (1.23 V / 1.739 V × 100%)**. In contrast, the symmetric NMF||NMF electrolyzer requires 2.244 V, corresponding to an energy efficiency of 54.81% (1.23 V / 2.244 V × 100%). The Pr‐doped device therefore achieves a 15.92 percentage‐point improvement in energy efficiency over the symmetric device.

**FIGURE 5:**
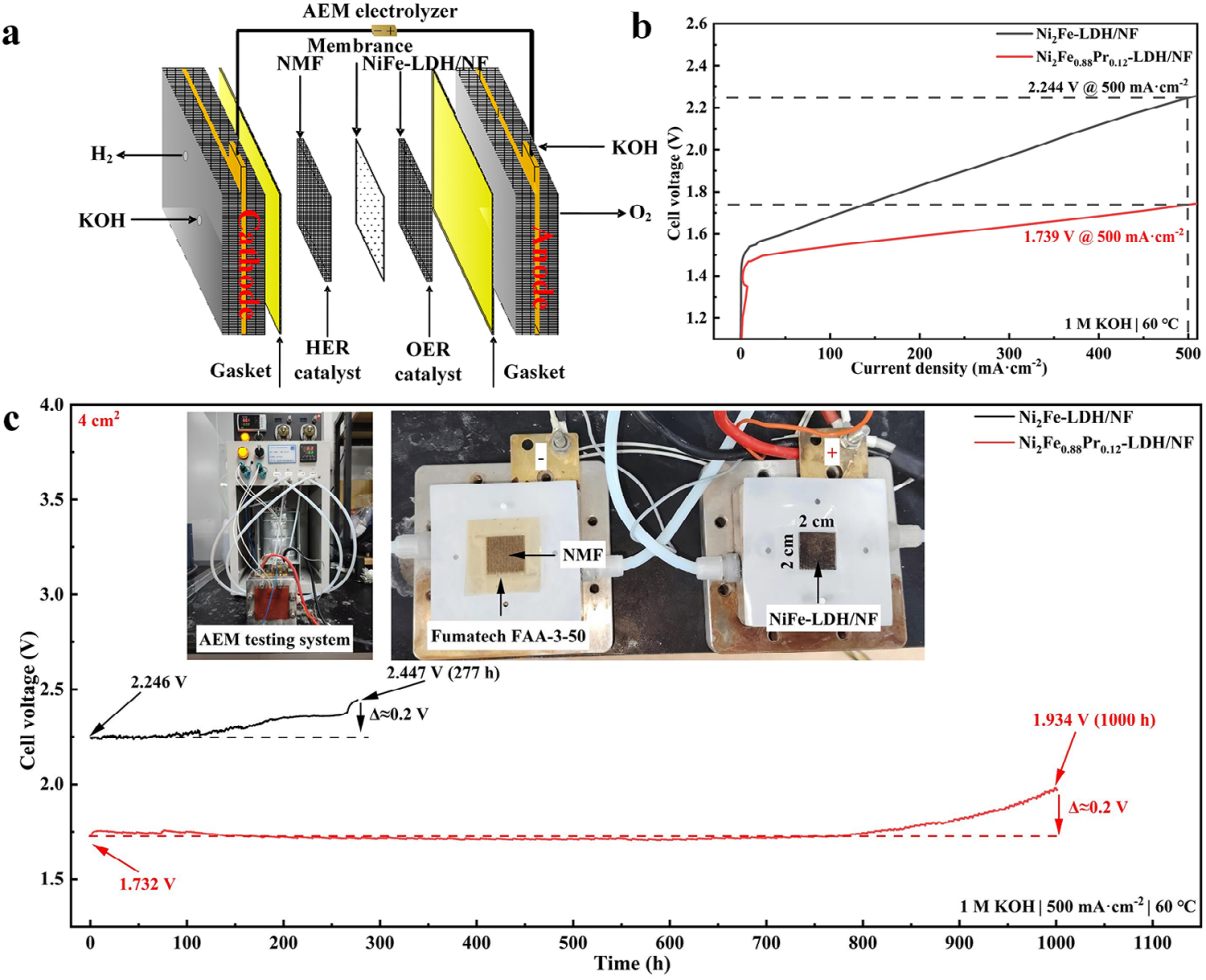
Electrochemical performance of the AEM electrolyzer with Ni_2_Fe_0.88_Pr_0.12_‐LDH anode. (a) Schematic diagram of MEA configuration. (b) Polarization curves of the AEM electrolyzers recorded without iR correction (as‐measured cell voltage). (c) Long‐term chronopotentiometric durability test.

Long‐term stability was evaluated at 500 mA cm^−2^ (Figure 5c). The NMF||Ni_2_Fe_0.88_Pr_0.12_‐LDH device operates for over 1000 h, with cell voltage increasing gradually from 1.732 to 1.934 V. This corresponds to a voltage drift of 0.202 V and an average degradation rate of ∼0.20 mV h^−1^. well below the commonly adopted industrial failure threshold for alkaline membrane electrolyzers (ΔV < 0.5 V). In contrast, the NMF||Ni_2_Fe‐LDH device reaches a 0.2 V drift after only 277 h, with the cell voltage rising from 2.246 to 2.447 V (Figure 5c). The >1000 h lifetime of the Pr‐doped electrolyzer surpasses most reported AEM systems (<800 h) and satisfies the industrial durability standard (≥800 h) [52, 53, 54]. Post‐mortem characterization after 1000 h AEM operation confirms no catastrophic degradation of the anode catalyst (Figures S9 and S10). TEM shows preserved ultrathin nanosheet morphology; STEM elemental mapping demonstrates homogeneous Ni, Fe, Pr, O and C distribution; HRTEM shows lattice fringes of 0.249 and 0.145 nm indexed to (012) and (113); SAED confirms crystallinity; XRD validates LDH phase stability; and XPS confirms stable Ni^2+^, Fe^3+^, Pr^3+^, O 1s, and C 1s states. ICP‐OES (Table S5) after 1000 h AEM shows Ni, Fe, and Pr at 97.1634, 1.2495, and 0.0622 wt.%, respectively. Based on a catalyst loading of ∼1.77 mg cm^−2^, Fe loss is ∼3.69 µg cm^−2^ and Pr loss is ∼0.363 µg cm^−2^ after 1000 h. Pr content decreases slightly, whereas Ni and Fe remain essentially constant, indicating excellent compositional stability under practical AEM operation. A performance comparison with recently reported advanced OER catalysts is provided in Table S6 [49, 55, 56, 57, 58, 59, 60, 61, 62, 63, 64, 65, 66, 67, 68, 69]. The comparison highlights the competitive activity and durability of Ni_2_Fe_0.88_Pr_0.12_‐LDH/NF at both the half‐cell and practical AEM electrolyzer levels, demonstrating its potential for industrial water electrolysis.

## 3 Conclusion

In summary, we have developed a Pr‐induced dual electronic and pore engineering strategy to boost the industrial OER performance of NiFe‐LDH. This work uniquely integrates atomic‐scale electronic redistribution (downshifting the d‐band center and enhancing Ni**─**O covalency) with nanoscale pore architecture refinement (increasing BET surface area by 13.4% and narrowing pore size by 12.8%). We demonstrate that **atomically dispersed Pr** ^3+^ **enlarges the LDH interlayer spacing (0.76 nm → 0.81 nm) and constructs a hierarchical nanoflower structure with refined mesopores**: the BET surface area increases by 13.4%, average BJH desorption pore size decreases by 12.8%, and pore volume/surface area are both improved, which accelerates mass transport and active site exposure. Mechanistically, strong Pr**─**O interactions trigger charge redistribution, enrich electron density on Ni sites, downshift the Ni 3d band center and enhance Ni**─**O covalency (ICOHP: −2.873 eV → −2.938 eV). A cascade charge‐transfer pathway (Pr → Fe → Ni via Pr**─**O**─**Fe**─**O**─**Ni bridges) is proposed to reconcile the XPS, XANES, and DFT results. These electronic modulations switch the OER RDS from ^*^O → ^*^OOH (0.74 eV) to ^*^OH → ^*^O (0.28 eV), drastically reducing the reaction barrier. Consequently, the optimized Ni_2_Fe_0.88_Pr_0.12_‐LDH delivers **407 mV overpotential at 500 mA cm^−2^ and 2190 h half‐cell durability**. When integrated into an AEM electrolyzer, it achieves >1000 h stable operation at industrial current densities with an average degradation rate of ∼0.20 mV h^−1^, fully satisfying industrial application requirements. More importantly, this is the first demonstration of an AEM electrolyzer with a rare‐earth‐doped LDH anode operating stably over 1000 h, filling the gap between fundamental material design and industrial‐scale water electrolysis. This work provides a universal RE modulation strategy for designing high‐performance, durable LDH‐based electrocatalysts, and offers new insights for the development of practical water electrolysis catalysts.

## 4 Experimental Section

Detailed experimental procedures, including synthesis, characterization, electrochemical measurements, XAFS analysis, DFT calculations, and AEM electrolyzer assembly, are provided in the Supporting Information.

## Author Contributions

**Yuanming Gao**: Writing – original draft, funding acquisition. **Xuezhen Wu**: writing – original draft. **Lin Zhang**: investigation. **Haoxiang Zhang**: investigation. **Xueying Feng**: investigation. **Yu Bai**: investigation. **Wei Song**: investigation. **Yong Zheng**: investigation. **Jiong Liu**: investigation. **Jieyu Chen**: investigation. **Dong Yang**: investigation, funding acquisition. **Wen Ma**: writing – review and editing, funding acquisition. **Shengzhong (Frank) Liu**: writing – review and editing, funding acquisition.

## Conflicts of Interest

The authors declare that they have no known competing financial interests or personal relationships that could have appeared to influence the work reported in this paper.

## Supporting information




**Supporting File**: advs78238‐sup‐0001‐SuppMat.docx.

## Data Availability

The data that support the findings of this study are available from the corresponding author upon reasonable request.
