# Supplementary material for: Praseodymium‐Modulated NiFe Layered Double Hydroxide with Enhanced Oxygen Evolution Activity and Extended Durability
Source: Adv Sci (Weinh). 2026 Oct 12:e78238. Online ahead of print. doi: 10.1002/advs.78238 (PMC13658888; doi:10.1002/advs.78238)
Supplement: Supplementary file 1 [file ADVS-9999-e78238-s001.docx]

**Experimental Section**

**Materials and Chemicals.** Ni(NO_3_)_2_·6H_2_O (≥ 98wt.%), Fe(NO_3_)_3_·9H_2_O (≥ 98wt.%), Pr(NO_3_)_3_·6H_2_O (≥ 99.9wt.%) were purchased from Shanghai Titan Scientific Co., Ltd., Shanghai, China, urea (≥ 99wt.%), NH_4_F (≥ 98wt.%), and KOH (≥ 95wt.%) were purchased from Shanghai Aladdin Biochemical Technology Co., Ltd., Shanghai, China. All reagents were used as received without further purification. Nickel foam was pretreated by sonication in 3 M HCl, acetone, ethanol, and deionized water sequentially, followed by drying at 60 °C overnight. A Fumasep FAA-3-50 membrane was pre-treated in 1.0 M KOH at room temperature for 24 h prior to use.

**Synthesis of Ni_2_Fe_0.88_Pr_0.12_-LDH and Control Samples.** In a typical synthesis, Ni(NO_3_)_2_·6H_2_O (10 mM), Fe(NO_3_)_3_·9H_2_O (5 mM), and Pr(NO_3_)_3_·6H_2_O (0.15–0.75 mM) were dissolved in 30 mL of deionized water under vigorous stirring, followed by the addition of urea (30 mM) and NH_4_F (20 mM). The pH of the mixed solution was adjusted to approximately 6.5 using dilute KOH. The resulting solution was transferred to a 50 mL Teflon-lined stainless-steel autoclave containing a piece of pretreated nickel foam and heated at 120 °C for 12 h. After natural cooling to room temperature, the sample was washed with deionized water and ethanol several times and dried at 60 °C overnight. For comparison, NiFe-LDH was synthesized under identical conditions without the addition of Pr(NO_3_)_3_·6H_2_O. The Pr-doped samples with different feed ratios are denoted as Ni_2_Fe_0.88_Pr_0.12_-LDH unless otherwise stated.

**Characterization.** XRD patterns were recorded on a Bruker D8 Advance diffractometer (D8 AXS, Germany) with Cu Kα radiation (λ = 1.5406 Å) at a scan rate of 10° min^-1^ over a 2θ range of 5–90. Raman spectra were collected on a LabRAM HR Evolution spectrometer (Horiba, Japan) using a 532 nm laser with an incident power of 5 mW. XPS measurements were performed on ESCALAB 250Xi spectrometer (Thermo Fisher Scientific, USA) using Al-Kα radiation (1486.6 eV); the binding energies were calibrated to the C 1s peak at 284.8 eV. SEM (Hitachi S-4800, Hitachi, Japan), TEM (Tecnai G2 F20 S-TWIN, Thermo Fisher Scientific, USA), HRTEM and HAADF-STEM (Spectra 300, Thermo Fisher Scientific, USA), and EDS (Super-X G2, Thermo Fisher Scientific, USA) were conducted. ICP-OES (Agilent 5110, Agilent Technologies, USA) was used to determine the Ni/Fe/Pr molar ratios after acid digestion in aqua regia. BET surface areas were measured with a MicrotracBEL analyzer (Japan) by N_2_ adsorption-desorption at 77 K after degassing at 60°C for 12 h.

**Electrochemical Measurements.** Electrochemical tests were performed on a CS350M workstation (Wuhan CorrTest Instruments Corp., Ltd., Wuhan, China) in a standard three-electrode cell with 1.0 M KOH aqueous solution as the electrolyte. The as-prepared sample on nickel foam (1 × 1 cm^2^), a graphite rod, and a Hg/HgO electrode were used as the working, counter, and reference electrodes, respectively. All potentials were converted to the reversible hydrogen electrode (RHE) scale using the equation:

E_RHE_ = E_ref_ + 0.059 × pH + $\text{E}_{\text{ref}}^{\text{o}}$. $\text{E}_{\text{ref}}^{\text{o}}$ = 0.098 V (Hg/HgO,1.0 M KOH), iR correction was performed using E_corr_ = E_meas_ − 0.95 × iR_s_, where R_s_ was determined by EIS at open circuit. LSV curves were recorded at a scan rate of 5 mV s^-1^ with 95% iR compensation. EIS was measured at OCP vs. RHE over a frequency range of 100 kHz to 0.1 Hz with an amplitude of 5 mV. The C_dl_ was obtained from CV curves at different scan rates of 20, 40, 60, 80, 100 mV s^-1^), and the ECSA was calculated as ECSA = C_dl_/C_s_. Stability tests were carried out by chronopotentiometry at 500 mA cm^-2^ for over 2000 h.

**AEM Electrolyzer Assembly and Testing.** The MEA was fabricated with a Ni_2_Fe_0.88_Pr_0.12_-LDH anode (effective area: 4 cm^2^), a commercial Ni-Mo foam cathode, and a Fumasep FAA-3-50 anion exchange membrane. The anode catalyst loadings were 1.77 mg cm^-2^. The electrolyzer was tested at 60 °C with 1.0 M KOH circulating at a flow rate of 40 mL min^-1^. Polarization curves and EIS data were recorded, and the stability was evaluated at a constant current density of 500 mA cm^-2^ for over 1000 h. No iR correction was applied to the AEM electrolyzer polarization curves. All cell voltages and energy efficiencies reported for the AEM electrolyzer are based on as-measured, non-iR-corrected values. The energy efficiency was calculated as EE = 1.23 V / V_cell_ (as-measured) × 100%.

**XAFS Measurements and Analysis.** XAFS data were collected at the BL14W1 beamline of the Shanghai Synchrotron Radiation Facility (SSRF). The Ni and Fe K-edge spectra were measured in transmission mode, and the Pr L_3_-edge spectrum was measured in fluorescence mode. Data processing was performed using ATHENA and ARTEMIS software. The *k*^3^-weighted EXAFS spectra were Fourier transformed in the *k*-range of 3.0–10.0 Å^-1^. Multi-shell fitting was performed to describe the local coordination environment, and the fitting parameters (k-range, R-range, N, R, σ**^2^**, ΔE_0_, and R-factor) are summarized in **Table S3**. WT analysis was performed using the Morlet wavelet with η=10 and σ=1. The R range was set from 1 to 5 Å and the k range from 1 to 15 Å^-1^ with a step size of 0.05 Å^-1^. It should be noted that the Ni–O and Fe–O bond lengths are reported in Å, not nm. The asymmetric EXAFS peak at approximately 2 Å for Ni_2_Fe_0.88_Pr_0.12_-LDH is discussed in the main text in terms of the limited *k*-range (*k*_max_ = 12 Å^-1^) and the presence of multiple coordination bonds, as supported by the WT results.

**DFT Calculations.** Spin-polarized DFT calculations were performed using the Vienna Ab initio Simulation Package (VASP) with the PBE+U functional (U_Ni_ = 6.0 eV, U_Fe_ = 4.0 eV). The plane-wave cutoff energy was 450 eV, and the Brillouin zone was sampled using a 3×3×1 Monkhorst-Pack *k*-point grid. A vacuum layer of [value] Å was used to avoid periodic interactions. All structures were fully relaxed until the forces were <0.02 eV Å^-1^. The adsorption energy was calculated as E_ads_ = E_total_ − E_slab_ − E_adsorbate_. Free energy corrections, including zero-point energy and entropy, were applied where necessary.

**Figure S1.** Microstructure and phase composition of Ni_2_Fe_1-x_Pr_x_-LDH/NF with various Pr doping contents (x=0.03, 0.06, 0.09, 0.12, 0.15). (a, b, c) SEM images recorded at different magnifications. (d, e, f) TEM images at different magnifications. (g) SAED pattern. (h, i) Representative HAADF-STEM image with the analyzed spots marked (some of the spots are highlighted by red circles). (j) Size distribution histogram of over 60 randomly selected bright spots from HAADF-STEM images. (k) Full-range XRD patterns. (l) Magnified view of selected XRD regions. (m, n) HAADF-STEM-EDS elemental mapping.

**Figure S1a-c** shows SEM images at sequential magnifications to characterize the macroscopic morphology of the catalysts. At low magnification (**Figure S1a**), the active material uniformly covers the skeleton of nickel foam. Medium-magnification SEM (**Figure S1b**) reveals interconnected nanosheet networks grown on the substrate. High-magnification SEM (**Figure S1c**) further verifies the well-defined two-dimensional sheet-like morphology of LDH. **Figure S1d-f** presents TEM images at different magnifications, displaying the typical ultrathin nanosheet structure of LDH. The nanosheets possess uniform lateral size and smooth surface, and no obvious agglomeration is observed for moderately doped samples. The corresponding SAED pattern in **Figure S1g** verifies high crystallinity and lattice expansion after Pr doping.

**Figure S1h, i** are representative HAADF-STEM images with bright atomic spots highlighted by red circles. Statistical analysis of these bright spots in **Figure S1j** reveals that >92% of the spots exhibit a size distribution centered at 0.21 ± 0.03 nm. This value matches the expected imaging contrast size of Pr^3+^ (ionic radius ~0.099 nm) substituting for Ni^2+^/Fe^3+^ sites in the LDH lattice and is markedly smaller than the typical imaging size of PrO_x_ nanoclusters (>0.5 nm). Combined with the absence of Pr–Pr scattering in EXAFS (**Figure 2i**) and the Pr–O coordination number of ~6.2 (consistent with the octahedral coordination environment within LDH layers), these results confirm that Pr is atomically dispersed as substitutional dopants within the LDH lattice, rather than surface-adsorbed species or PrO_x_ clusters.

XRD was performed to analyze the crystal phase of Pr-doped NiFe-LDH grown on nickel foam. The full-range XRD patterns in **Figure S1k** show all diffraction peaks can be well assigned to the standard LDH phase (PDF#00-051-0463, Ni_5.64_Fe_2.36_(OH)_16_(CO_3_)_1.18_·7.52H_2_O) and metallic Ni substrate (PDF#00-004-0850). The magnified XRD profile in **Figure S1l** clearly presents the characteristic diffraction features of LDH. No impurity signals are detected for samples with x ≤ 0.12, confirming pure LDH phase. Weak peaks corresponding to Pr_2_O_3_ appear at x = 0.15, demonstrating the segregation of Pr species at excessive doping levels. **Figure S1m, n** shows HAADF-STEM-EDS elemental mapping results, which display homogeneous distribution of Ni, Fe, Pr, O, C and N across the entire nanosheet, confirming uniform Pr doping and excellent structural integrity. Collectively, **Figure S1a-n** verify the successful fabrication of Pr-doped NiFe-LDH with high crystallinity, regular morphology and homogeneous elemental distribution.

**Table S1.** ICP-OES elemental analysis of as-prepared Ni_2_Fe_1-x_Pr_x_-LDH/NF catalysts (x=0.03, 0.06, 0.09, 0.12, 0.15) and pristine Ni_2_Fe-LDH/NF.

| Sample | Ni/wt.% | Fe/wt.% | Pr/wt.% | Balance/wt.% |
| --- | --- | --- | --- | --- |
| Ni_2_Fe_0.97_Pr_0.03_-LDH/NF | 96.4332 | 1.5451 | 0.0281 | 1.9936 |
| Ni_2_Fe_0.94_Pr_0.06_-LDH/NF | 96.3398 | 1.4951 | 0.0446 | 2.1205 |
| Ni_2_Fe_0.91_Pr_0.09_-LDH/NF | 96.9240 | 1.4884 | 0.0648 | 1.5228 |
| Ni_2_Fe_0.88_Pr_0.12_-LDH/NF | 96.8030 | 1.4581 | 0.0827 | 1.6562 |
| Ni_2_Fe_0.85_Pr_0.15_-LDH/NF | 96.6963 | 1.4552 | 0.0838 | 1.7647 |
| Ni_2_Fe-LDH | 96.5832 | 1.5234 | N/A | 1.8934 |

All elemental contents are reported in weight percent (wt.%). **Table S1** lists the ICP‑OES elemental mass fractions of fresh pristine and gradient Pr‑doped Ni_2_Fe‑LDH/NF catalysts. The measured Pr content gradually increases with the nominal Pr‑doping fraction. When the nominal doping reaches (x=0.15), the growth of detected Pr becomes very limited, indicating that the LDH lattice approaches its Pr‑doping saturation limit. The mass fractions of Ni and Fe remain relatively stable across all samples, demonstrating that the host NiFe‑LDH crystal framework is well preserved during Pr incorporation. The “Balance” term accounts for light elements (O, C, H, etc.) that cannot be quantified by ICP‑OES.

**Figure S2.** (a) Optimized structural models of OER intermediates on catalyst surface of Ni_2_Fe_0.88_Pr_0.12_-LDH. (b–e) Pore structure characterization of Ni_2_Fe-LDH and Ni_2_Fe_0.88_Pr_0.12_-LDH: (b) BJH cumulative pore volume. (c) BJH cumulative surface area. (d) N_2_ adsorption-desorption isotherms. (e) BJH desorption pore size distribution.

**Figure S2a** shows the fully optimized atomic structural models of three key OER reaction intermediates (*OH, *O, *OOH) adsorbed on the surface of pristine Ni_2_Fe-LDH and Ni_2_Fe_0.88_Pr_0.12_-LDH. The atom color coding is as follows: H (pale pink), O (red), Ni (light gray), Fe (orange-brown), Pr (lavender purple). For both catalysts, Ni sites serve as the dominant adsorption sites for all intermediates. After Pr atomic incorporation into the LDH lattice, the local lattice undergoes noticeable expansion, and the bonding distances between Ni active sites and adsorbed intermediates are subtly modulated by strong Pr–O electronic coupling. These geometric and electronic modifications at the active interface directly adjust the Gibbs adsorption free energies of *OH, *O, and *OOH species, thereby optimizing the OER reaction pathway and lowering the energy barrier of the rate-determining step.

The porous structure of the two catalysts was further characterized by N_2_ adsorption-desorption measurements. The BJH cumulative pore volume (**Figure S2b**) of Pr-doped LDH increases by 4.8% compared with the pristine sample, while the BJH cumulative surface area (**Figure S2c**) shows a remarkable enhancement of 20.1%. The N_2_ adsorption-desorption isotherms (**Figure S2d**) of both materials are identified as typical type IV isotherms with H3-type hysteresis loops in the relative pressure range of p/p_0_ = 0.4-1.0, indicating the mesoporous nature of the LDH materials. From the BJH desorption pore-size distribution histogram (**Figure S2e**), Pr doping narrows the overall pore-size distribution and increases the proportion of mesopores in the 2-5 nm range.

The optimized pore structure induced by Pr doping facilitates electrolyte infiltration and shortens the mass-transport pathways for OH^–^ and generated O_2_. Meanwhile, the enlarged specific surface area improves the accessibility of catalytically active sites. These structural benefits collectively contribute to the superior high-current-density OER performance and long-term stability of Ni_2_Fe_0.88_Pr_0.12_-LDH.

**Table S2.** Textural properties of pristine Ni_2_Fe-LDH and Pr-doped Ni_2_Fe_0.88_Pr_0.12_-LDH derived from N_2_ adsorption-desorption isotherms (77 K).

| Properties | Ni_2_Fe-LDH | Ni_2_Fe_0.88_Pr_0.12_-LDH |
| --- | --- | --- |
| BET surface area/m^2^·g^–1^ | 27.04 | 30.67 |
| Micropore volume/cm^3^ g^–1^ | 0.00001 | 0.00034 |
| Micropore area/m^2^ g^–1^ | 0.01 | 0.55 |
| Average pore size-BJH desorption/nm | 8.39 | 7.32 |
| Average pore size-BET desorption/nm | 25.89 | 22.96 |
| BJH desorption cumulative pore volume/cm^3^ g^–1^ | 0.2317 | 0.2429 |
| BJH desorption cumulative surface area/m^2^ g^–1^ | 110.50 | 132.70 |
| BET correlation coefficient | 0.9962 | 0.9989 |

*BET: Brunauer-Emmett-Teller; BJH: Barrett-Joyner-Halenda.*

Pr doping raises the BET surface area by 13.4%, reduces the average BJH desorption pore size by 12.8%, and increases the cumulative pore volume by 4.8%. These changes improve the accessibility of catalytic active sites and accelerate mass transport during the OER process.

**Figure S3.** XPS and WT-EXAFS characterizations of Ni_2_Fe_0.88_Pr_0.12_-LDH. (a) XPS survey spectra of Ni_2_Fe_1-x_Pr_x_-LDH with different Pr contents (x = 0.03, 0.06, 0.09, 0.12, 0.15). (b) High-resolution O 1s XPS spectra. (c) High-resolution C 1s XPS spectra. (d–f) K-space EXAFS oscillations of Ni K-edge, Fe K-edge and Pr L_3_-edge, respectively. (g–i) R-space EXAFS spectra (Fourier transforms) of Ni K-edge, Fe K-edge and Pr L_3_-edge, respectively.

XPS was employed to identify the elemental composition and surface chemical states of the as-prepared catalyst. The survey XPS spectrum (**Figure S3a**) presents distinct characteristic signals of Ni, Fe, Pr, O and C for all samples with different Pr contents, confirming the successful incorporation of all constituent elements in Pr-doped NiFe-LDH. With increasing Pr content, the intensity of the Pr 3d peaks increases accordingly, while the positions remain stable, indicating the uniform incorporation of Pr^3+^ without valence fluctuation.

The high-resolution O 1s spectrum (**Figure S3b**) can be deconvoluted into four main components centered at approximately 529.5 eV (M–O), 531.0 eV (M–OH), 532.0 eV (defect-related oxygen species) and 533.4 eV (carbonate species, O–C=O). With the increase of Pr doping content, the M–O peak gradually shifts toward lower binding energy, accompanied by a noticeable enhancement in the intensity of defect-related oxygen signals. These variations in peak position and relative intensity manifest the formation of strong Pr–O interactions and effective modulation of the surface oxygen electronic environment.

The high-resolution C 1s spectrum (**Figure S3c**) is deconvoluted into three characteristic peaks located at around 284.6 eV (C=C), 286.2 eV (C–O) and 288.8 eV (O–C=O). The O–C=O component originates from interlayer carbonate anions that maintain the charge balance of the LDH layered framework. Upon increasing Pr doping content, the O–C=O peak exhibits a slight shift toward lower binding energy, and the relative intensity of each carbon species changes correspondingly. This trend reveals that Pr doping subtly alters the electronic state of interlayer carbonate groups, while the intrinsic carbon configuration of the LDH structure is well preserved.

Synchrotron-based WT-EXAFS measurements were further carried out to investigate the local coordination environment of Ni, Fe and Pr in Ni_2_Fe_0.88_Pr_0.12_-LDH. The K-space EXAFS oscillations (**Figure S3d–f**) and the corresponding R-space spectra (**Figure S3g–i**) show characteristic coordination peaks for Ni–O (~1.62 Å), Fe–O (~1.60 Å) and Pr–O (~2.00 Å). Besides the inherent Ni–Ni and Fe–Ni coordination peaks, no additional peaks corresponding to Pr–Pr scattering or metal-metal agglomeration are observed, further verifying the atomic dispersion of Ni, Fe and Pr atoms within the LDH lattice. All results collectively validate the well-maintained local atomic structure of LDH and the uniform doping of Pr heteroatoms.

**Figure S4.** EXAFS and WT-EXAFS characterizations of pristine Ni_2_Fe-LDH (control sample). (a, d) WT-EXAFS contour plots of Ni K-edge and Fe K-edge. (b, e) K-space EXAFS oscillations and (c, f) corresponding R-space EXAFS spectra. Compared with the Pr-doped sample (Figure S2), pristine Ni^2^Fe-LDH shows shorter Ni–O/Fe–O bonds, more symmetric R-space peaks, and no Pr–O coordination signal.

To clarify the effect of Pr doping on the local coordination environment, EXAFS and WT-EXAFS analyses were also performed on pristine Ni_2_Fe-LDH as a control (**Figure S4**) and compared with Ni_2_Fe_0.88_Pr_0.12_-LDH (**Figure S3**).

The pristine Ni₂Fe-LDH exhibits characteristic Ni–O and Fe–O peaks at ~1.59 Å and ~1.60 Å, respectively, with symmetric R-space features and no high-R scattering signals. After Pr doping, the Ni–O bond length increases to ~1.618 Å and the Fe–O bond length slightly increases to ~1.603 Å, confirming the lattice expansion induced by the larger Pr^3+^ ion. Meanwhile, the R-space peaks become noticeably asymmetric around 1.5–2.0 Å, which is attributed to multiple Ni/Fe–O coordination environments created by Pr–O–M bridges. In addition, a distinct Pr–O coordination peak at ~2.00 Å appears in the Pr-doped sample, while no Pr–Pr scattering is detected, further confirming the atomic-level dispersion of Pr within the LDH lattice.

These comparisons demonstrate that Pr incorporation not only expands the interlayer spacing but also modulates the local coordination environment of Ni and Fe sites, consistent with the enhanced OER activity and the switched rate-determining step observed in our DFT calculations. The complete EXAFS fitting parameters are summarized in Table S3.

**Table S3.** Ni and Fe K-edge EXAFS fitting results

| **Sample** | **Ni_2_Fe-LDH** | | | | | **Ni_2_Fe_0.88_Pr_0.12_-LDH** | | | |
| --- | --- | --- | --- | --- | --- | --- | --- | --- | --- |
| **Path** | **Fe-O** | **Fe-M** | **Ni-O** | **Ni-M** | **Fe-O** | | **Fe-M** | **Ni-O** | **Ni-M** |
| **K-range/Å^–1^** | 3.0-10.0 | | | | | 3.0-10.0 | | | |
| **R-range/Å** | 1.0-3.18 | | | | | 1.0-2.65 | | | |
| ***N*** | 6.836±1.52 | 3.822±2.21 | 6.878±0.98 | 3.723±0.76 | 6.937±0.17 | | 4.030±0.73 | 6.919±0.71 | 3.850±2.77 |
| ***R*/Å** | 2.00023±0.019 | 3.01677±0.019 | 2.06026±0.009 | 3.05604±0.009 | 2.011±  0.016 | | 3.039±  0.016 | 2.074±0.05 | 3.078±0.05 |
| ***σ*^2^×10^3^**  **/Å^2^** | 6.61±3.74 | 6.32±3.74 | 8.13±1.80 | 6.32±1.80 | 7.12±4.41 | | 6.16±8.40 | 9.25±1.60 | 7.84±1.60 |
| **Δ*E* /eV** | -2.701±2.47 | -2.701±2.47 | -2.846±1.05 | -2.846±1.05 | -8.316±3.88 | | -8.316±3.88 | -8.706±1.05 | -8.706±1.05 |
| ***R*-factor** | 0.013 | 0.013 | 0.005 | 0.005 | 0.003 | | 0.003 | 0.011 | 0.011 |

*N*: coordination numbers; *R*: bond distance; *σ*^2^: Debye-Waller factors; Δ*E*: the inner potential correction. *R* factor: goodness of fit. Fitting with fixed parameter. The number of variable parameters is 7.

**Figure S5.** Electrochemical OER performance of Ni_2_Fe-LDH/NF synthesized under different hydrothermal conditions. (a–d) iR-corrected LSV curves. (e–h) statistical overpotentials. (i–l) corresponding Tafel plots for samples prepared at 100 °C, 120 °C, 140 °C and 160 °C with reaction durations of 6, 8, 10 and 12 h, respectively. All electrochemical tests were performed in 1.0 M KOH electrolyte.

To identify the optimal hydrothermal parameters for Ni_2_Fe-LDH synthesis, we systematically varied the hydrothermal temperature (100, 120, 140, 160 °C) and reaction duration (6, 8, 10, 12 h). The overpotentials at 10, 50, and 100 mA cm^-2^ were selected as key metrics to evaluate OER catalytic activity. At 100 °C, the hydrothermal reaction proceeds incompletely, yielding products with poor crystallinity and large overpotentials (e.g., 365 mV at 10 mA cm^-2^ for the 12 h sample). Increasing the temperature to 120 °C substantially improves the crystallinity of Ni_2_Fe-LDH and exposes more electrochemically active sites. Among this series, the sample synthesized at 120 °C for 12 h achieves the lowest overpotential of 198 mV at 10 mA cm^-2^. Further elevation of the temperature to 140 °C triggers excessive crystal growth and partial phase segregation, which increases the required overpotential. At 160 °C, severe particle agglomeration together with the formation of inactive NiO impurities further degrades the OER performance.

Tafel slopes extracted from the iR-corrected LSV curves were analyzed to probe the OER reaction kinetics. The trend of Tafel slopes agrees well with the overpotential variation across all hydrothermal conditions. The catalyst prepared at 120 °C for 12 h delivers the smallest Tafel slope of 63.27 mV dec^-1^, indicative of the fastest OER kinetics. Samples synthesized at 100 °C exhibit Tafel slopes above 80 mV dec^-1^, arising from incomplete crystallization and sluggish interfacial charge transfer. For the 140-160 °C series, Tafel slopes increase again, primarily due to reduced electrochemically active surface area and elevated interfacial resistance induced by structural defects and particle aggregation.

Collectively, the LSV profiles, overpotential statistics and Tafel analysis confirm that 120 °C and 12 h represent the optimal hydrothermal conditions. This parameter set achieves a favorable balance among crystallinity, defect density and active-site exposure. Unless otherwise noted, all subsequent catalysts in this work were fabricated using this optimized hydrothermal recipe.

**Figure S6.** Effect of Ni:Fe atomic ratio on the OER performance of Ni_x_Fe-LDH catalysts. (a) iR-corrected LSV curves recorded at 5 mV s^-1^ in 1.0 M KOH. (b) Tafel slopes. (c) EIS Nyquist plots with equivalent circuit model. Statistical overpotentials at 10, 50 and 100 mA cm^-2^ (d-f). C_dl_ derived from CV measurements (g-i). (d, g) Ni_2_Fe_0.91_RE_0.09_-LDH (RE = La, Ce, Pr, Nd). (e, h) Ni_2_Fe_1-x_Pr_x_-LDH, (f, i) Ni_x_Fe-LDH. (j) Bode plots of pristine Ni_2_Fe-LDH and Ni_2_Fe_0.88_Pr_0.12_-LDH derived from EIS measurements at 1.55 V vs. RHE. (k) LSV curves normalized by ECSA and (l) LSV curves at 500 mA cm^-2^ of Ni_2_Fe_0.88_Pr_0.12_-LDH and Ni_2_Fe-LDH.

The Ni/Fe atomic ratio plays a decisive role in the OER activity of NiFe‑LDH by tuning the electronic configuration of Ni sites via Fe modulation. A series of Ni_x_Fe‑LDH (Ni:Fe = 2:1, 3:1, 4:1) were prepared with fixed total metal content to screen the optimal stoichiometry. **Figure S6a** displays the iR-corrected LSV profiles, where Ni_2_Fe-LDH delivers the best catalytic performance. The corresponding overpotential statistics in **Figure S6f** verify that Ni_2_Fe-LDH achieves 225 mV @10 mA cm^-2^ and 379 mV @100 mA cm^-2^, outperforming Ni_3_Fe-LDH and Ni_4_Fe-LDH. **Figure S6b** presents Tafel-slope comparisons; Ni_2_Fe-LDH shows the smallest Tafel slope of 52.3 mV dec^-1^, reflecting accelerated OER kinetics. EIS Nyquist plots in **Figure S6c** demonstrate the lowest charge-transfer resistance for Ni_2_Fe-LDH, indicative of favorable interfacial charge transport. The C_dl_ results in **Figure S6i** reveal that Ni_2_Fe-LDH possesses the largest electrochemical active surface area among the Ni_x_Fe-LDH series. The electrochemically active surface area (ECSA) was estimated from C_dl_ using:

$\text{ECSA}\text{=}\frac{\text{C}_{\text{dl}}}{\text{C}_{\text{s}}}$ Supporting formula 1
Where C_s_ = 0.04 mF cm^-2^ based on values reported for metal electrodes in aqueous alkaline solution, following established protocols. The C_dl_ values were obtained from non-Faradaic CV curves at scan rates of 20, 40, 60, 80, and 100 mV s^-1^ in the non-Faradaic potential region (**Figure S6g-i**). Collectively, **Figure S6a-c,f,i** confirm the Ni:Fe=2:1 as the preferred baseline composition for subsequent rare-earth modification.

Four rare‑earth elements (La, Ce, Pr, Nd) were screened at identical doping content. From the overpotential statistics in **Figure S6d** and C_dl_ values in **Figure S6g**, Pr‑doped Ni_2_Fe‑LDH exhibits the lowest overpotential and maximum C_dl_ (237.74 mF cm^-2^), superior to La‑, Ce‑ and Nd‑doped counterparts, confirming Pr as the optimal dopant. Gradient Pr‑doped Ni_2_Fe_1-x_Pr_x_‑LDH were further investigated. **Figure S6e** summarizes the overpotential statistics of Ni_2_Fe_1-x_Pr_x_‑LDH, and **Figure S6h** gives the corresponding Cdl evolution. Both datasets identify Ni_2_Fe_0.88_Pr_0.12_‑LDH (x = 0.12) as the optimal sample; further Pr addition (x = 0.15) triggers impurity segregation and performance decay.

**Figure S6j** compares Bode spectra of pristine Ni_2_Fe-LDH and Ni_2_Fe_0.88_Pr_0.12_-LDH, consistent with improved charge‑transfer capability after Pr incorporation. **Figure S6k** shows ECSA‑normalized LSV curves, proving the intrinsic catalytic activity enhancement originating from Pr doping rather than merely surface‑area contribution. **Figure S6l** provides high‑current LSV data: Ni_2_Fe_0.88_Pr_0.12_-LDH only requires 1.637 V vs. RHE to reach 500 mA cm^-2^ (η = 407 mV), in sharp contrast to 2.169 V vs. RHE for undoped Ni_2_Fe‑LDH under identical industrial‑relevant current density.

**Table S4.** Overpotential statistics at 10-500 mA cm^-2^ of Ni_2_Fe_0.88_Pr_0.12_-LDH and Ni_2_Fe-LDH.

| Sample | 10 mA cm^-2^ | 50 mA cm^-2^ | 100 mA cm^-2^ | 200 mA cm^-2^ | 300 mA cm^-2^ | 400 mA cm^-2^ | 500 mA cm^-2^ |
| --- | --- | --- | --- | --- | --- | --- | --- |
| Ni_2_Fe-LDH/NF | 225 | 300 | 379 | 524 | 663 | 800 | 939 |
| Ni_2_Fe_0.88_Pr_0.12_-LDH/NF | 198 | 230 | 256 | 296 | 335 | 366 | 407 |

**Table S4** summarizes the overpotential values of Ni_2_Fe-LDH/NF and Ni_2_Fe_0.88_Pr_0.12_-LDH/NF across a wide current density range from 10 to 500 mA cm^-2^. At 10 mA cm^-2^, the Pr-doped catalyst delivers an overpotential of 198 mV, which is lower than the 225 mV of pristine Ni_2_Fe-LDH/NF. The performance gap becomes much more pronounced as current density increases. At the industrial-relevant current density of 500 mA cm^-2^, Ni_2_Fe_0.88_Pr_0.12_-LDH/NF only requires an overpotential of 407 mV, while Ni_2_Fe-LDH/NF needs 939 mV. These data quantitatively demonstrate that Pr doping substantially reduces the OER overpotential and maintains superior catalytic activity under high-current operating conditions.

**Figure S7.** Structural, morphological and compositional stability of Ni_2_Fe_0.88_Pr_0.12_-LDH after 200 h OER durability test. (a,b) TEM images at different magnifications. (c) STEM elemental mapping of Ni, Fe, Pr, O and C. (d) Post-cycling HRTEM image. (e) Corresponding SAED pattern. (f) XRD patterns before and after the stability test.

A series of characterizations were performed to assess the structural robustness of Ni_2_Fe_0.88_Pr_0.12_-LDH after long‑term OER electrolysis. TEM images at different magnifications (**Figure S7a,b**) show that the intrinsic ultrathin nanosheet morphology of the LDH is well preserved, with no obvious structural collapse or particle agglomeration. STEM elemental mapping (**Figure S7c**) demonstrates the homogeneous distribution of Ni, Fe, Pr, O and C over the entire catalyst, and no detectable segregation of Pr species occurs. The post‑test HRTEM image (**Figure S7d**) displays well‑resolved lattice fringes with interplanar spacings of 0.249 nm and 0.145 nm, which can be indexed to the (012) and (113) crystal planes of the LDH phase, respectively. The SAED pattern (**Figure S7e**) exhibits clear diffraction rings, confirming retention of satisfactory crystallinity after 200 h continuous electrolysis.

XRD patterns (**Figure S7f**) further validate the crystal‑phase stability. The characteristic LDH diffraction peaks corresponding to the (003), (006) and (012) facets remain well maintained after the durability test and match the standard PDF card (00‑051‑0463). No diffraction signals assigned to Pr_2_O_3_ or NiO impurities are observed, which excludes phase transformation or secondary impurity formation during prolonged OER operation. Collectively, these results confirm that Ni_2_Fe_0.88_Pr_0.12_-LDH exhibits outstanding morphological, crystalline and compositional stability under long‑duration OER catalysis.

**Figure S8.** Post-OER XPS characterizations of Ni_2_Fe_0.88_Pr_0.12_-LDH/NF after 200 h OER durability test. (a) Full-range XPS survey spectrum. High-resolution XPS spectra of (b) Ni 2p. (c) Fe 2p. (d) Pr 3d. (e) O 1s. (f) C 1s.

Post-test XPS measurements were conducted to systematically evaluate the surface elemental composition and chemical-state stability of Ni_2_Fe_0.88_Pr_0.12_-LDHLDH/NF following 200 h continuous OER electrolysis. The full-range XPS survey spectrum (**Figure S8a**) presents distinct characteristic signals for Ni, Fe, Pr, O, and C, confirming that major constituent elements are retained without obvious surface leaching after long-term catalysis.

The high-resolution Ni 2p spectrum (**Figure S8b**) exhibits prominent peaks at ~855.6 eV (2p_3/2_) and ~873.4 eV (2p_1/2_), accompanied by evident shake-up satellite features. These observations verify the stable presence of Ni^3+^ originating from the active NiOOH phase, which serves as the dominant active site for OER. The well-maintained Ni^3+^ species guarantee sustained catalytic activity during prolonged electrolysis.

The high-resolution Fe 2p spectrum (**Figure S8c**) shows characteristic peaks located at ~712.1 eV (2p_3/2_) and ~724.2 eV (2p_1/2_), consistent with typical Fe^3+^ signatures. This suggests that Fe maintains its +3 valence state, and no noticeable redox transformation takes place upon long-term electrocatalysis.

The high-resolution Pr 3d spectrum (**Figure S8d**) displays two well-defined peaks at ~934.6 eV (3d_5/2_) and ~954.7 eV (3d_3/2_). The fixed binding-energy positions confirm that Pr remains in the +3 oxidation state after OER cycling, without valence‑state variation or elemental segregation.

Moreover, the high-resolution O 1s (**Figure S8e**) and C 1s (**Figure S8f**) spectra show nearly identical peak positions and deconvoluted sub-peak components compared with the fresh catalyst. The oxygen-containing moieties and interlayer carbonate groups of the LDH lattice are well preserved, indicating negligible modification of the surface chemical environment.

Collectively, the retained elemental composition and stable chemical states of Ni, Fe, Pr, O and C after 200 h OER operation demonstrate the favorable surface chemical stability of Ni_2_Fe_0.88_Pr_0.12_-LDH/NF, which underpins its superior long-term electrocatalytic durability.

**Table S5.** ICP-OES elemental analysis of pristine Ni_2_Fe-LDH/NF and Ni_2_Fe_0.88_Pr_0.12_-LDH/NF after 200 h half-cell OER operation and 1000 h AEM electrolyzer test.

| Sample | Ni/wt.% | Fe/wt.% | Pr/wt.% | Balance/wt.% |
| --- | --- | --- | --- | --- |
| Ni_2_Fe-LDH  (200 h-Half cell) | 97.1872 | 0.8234 | N/A | 1.9894 |
| Ni_2_Fe_0.88_Pr_0.12_-LDH  (200 h-Half cell) | 96.9845 | 1.4395 | 0.0785 | 1.4975 |
| Ni_2_Fe_0.88_Pr_0.12_-LDH  (1000 h-AEM electrolyzer) | 97.1634 | 1.2495 | 0.0622 | 1.5249 |

**Table S5** summarizes the post-stability-test elemental compositions obtained from ICP-OES measurements. After 200 h of half-cell OER operation, the pristine Ni_2_Fe-LDH/NF suffers severe Fe leaching: the Fe mass fraction decreases sharply from 1.5234 wt.% (fresh sample in **Table S1**) to 0.8234 wt.%. In comparison, Ni_2_Fe_0.88_Pr_0.12_-LDH/NF retains most of its Fe and Pr after the identical 200 h half-cell test. Even under harsher practical AEM electrolyzer conditions over 1000 h, the Pr-modified catalyst still maintains considerable Fe and Pr contents, with only moderate element dissolution observed. These ICP-OES results confirm that Pr doping effectively mitigates Fe dissolution under alkaline OER conditions and improves the compositional stability of NiFe-LDH catalysts. The “Balance” represents unmeasurable light-element components.

**Figure S9.** Structural, morphological and compositional stability of Ni_2_Fe_0.88_Pr_0.12_-LDH/NF after 1000 h operation in an AEM electrolyzer. (a,b) TEM images at different magnifications. (c) STEM elemental mapping of Ni, Fe, Pr, O and C. (d) Post‑cycling HRTEM image. (e) Corresponding SAED pattern. (f) XRD patterns collected before and after the 1000 h AEM electrolyzer stability test.

A series of characterizations were conducted to assess the structural robustness of Ni_2_Fe_0.88_Pr_0.12_-LDH/NF after ultra‑long‑term operation within an AEM electrolyzer. TEM images at different magnifications (**Figure S9a,b**) show that the intrinsic ultrathin nanosheet morphology of the LDH is well preserved, with no obvious structural collapse or particle agglomeration. STEM elemental mapping (**Figure S9c**) demonstrates the homogeneous distribution of Ni, Fe, Pr, O and C over the entire catalyst, and no detectable segregation of Pr species occurs. The post‑test HRTEM image (**Figure S9d**) displays well‑resolved lattice fringes with interplanar spacings of 0.249 nm and 0.145 nm, which can be indexed to the (012) and (113) crystal planes of the LDH phase, respectively. The SAED pattern (**Figure S9e**) exhibits clear diffraction rings, confirming retention of satisfactory crystallinity after 1000 h continuous electrolysis in the AEM electrolyzer.

XRD patterns (**Figure S9f**) further validate the crystal‑phase stability. The characteristic LDH diffraction peaks corresponding to the (003), (006) and (012) facets remain well maintained after the durability test and match the standard PDF card (00‑051‑0463). No diffraction signals assigned to Pr_2_O_3_ or NiO impurities are observed, which excludes phase transformation or secondary impurity formation during prolonged AEM electrolyzer operation. Collectively, these results confirm that Ni_2_Fe_0.88_Pr_0.12_-LDH/NF exhibits outstanding morphological, crystalline and compositional stability even under 1000 h continuous operation in an AEM electrolyzer.

**Figure S10.** Post-OER XPS characterizations of Ni_2_Fe_0.88_Pr_0.12_-LDH/NF after 1000 h operation in an AEM electrolyzer. (a) Full-range XPS survey spectrum. high-resolution XPS spectra of (b) Ni 2p. (c) Fe 2p. (d) Pr 3d. (e) O 1s. (f) C 1s.

To further evaluate the surface chemical stability under industrially relevant conditions, post-test XPS measurements were performed on the identical Ni_2_Fe_0.88_Pr_0.12_-LDH/NF catalyst after 1000 h of continuous operation in an AEM electrolyzer (**Figure S10**). In contrast to the 200 h OER durability test (**Figure S8**), the full-range survey spectrum (**Figure S10a**) still presents distinct signals for Ni, Fe, Pr, O and C, confirming that the major constituent elements are robustly retained without obvious surface leaching after prolonged device-level electrolysis.

The high-resolution Ni 2p spectrum (**Figure S10b**) exhibits prominent peaks at ~855.6 eV (2p_3/2_) and ~873.4 eV (2p_1/2_) with evident shake-up satellites, which is highly consistent with the 200 h-tested sample. This verifies the stable presence of Ni^3+^ species originating from the active NiOOH phase, ensuring sustained catalytic activity. Similarly, the Fe 2p spectrum (**Figure S10c**) shows characteristic peaks at ~712.1 eV (2p_3/2_) and ~724.2 eV (2p_1/2_), indicating that Fe maintains its +3 valence state without noticeable redox transformation.

For the Pr 3d spectrum (Figure S9d), two well-defined peaks at ~934.6 eV (3d_5/2_) and ~954.7 eV (3d_3/2_) confirm that Pr remains in the +3 oxidation state, with no valence-state variation or elemental segregation. Furthermore, the O 1s (**Figure S10e**) and C 1s (**Figure S10f**) spectra show nearly identical peak positions and deconvoluted components to those of the fresh and 200 h-tested samples, indicating that the oxygen-containing moieties (M-OH, M-O) and interlayer carbonate/surface functional groups (O-C=O, C-O, C-C) of the LDH lattice are well preserved.

Collectively, the retained elemental composition and stable chemical states of Ni, Fe, Pr, O, and C after 1000 h of AEM electrolyzer operation demonstrate the superior surface chemical stability of Ni_2_Fe_0.88_Pr_0.12_-LDH/NF, which fundamentally underpins its exceptional long-term electrocatalytic durability.

**Table S6.** Comparison of OER performance of Ni_2_Fe_0.88_Pr_0.12_-LDH with recently reported state‑of‑the‑art catalysts in alkaline media (1.0 M KOH, unless otherwise specified). *Abbreviations: η, overpotential; R_ct_, charge transfer resistance; C_dl_, double‑layer capacitance; h, hours; A, ampere; RT, room temperature.*

| Catalysts | Overpotential/mV (1.0 M KOH) | | Tafel slope/mV dec^-1^ | R_ct_/Ω | C_dl_/  mF cm^-2^ | Stability | iR compensation | Ref. |
| --- | --- | --- | --- | --- | --- | --- | --- | --- |
| NiFe LDH/FeMoO heterostructure | 289@1A cm^-2^ | 316@2A cm^-2^ | 48.9 | – | 3.52 | 3000 h@2 A cm^-2^ | 90% | [55] |
| RT-NiFe-LDH | – | 230@0.5A cm^-2^ | 36.22  (65 ºC) | 0.0898 | – | 100 h@1 A cm^-2^ | – | [56] |
| ACCE-a (NiFe-LDH/metallic Ni/and metallic /SS) | 232@0.01A cm^-2^ | 348@0.5A cm^-2^ | 42.1 | 0.42 | 3.84 | 1000 h@0.5 A cm^-2^ | 95% | [57] |
| NiFe-SQ/NF-R | 261@0.5A cm^-2^ | 284@1A cm^-2^ | 50 | 0.38±  0.02 | 99.75 | 700 h@3 A cm^-2^ | 95% | [58] |
| NiFe-LS | 195@0.01A cm^-2^ | 220@0.1A cm^-2^ | 35.93 | – | 9.12 | 500 h@200 mA cm^-2^ | 90% | [59] |
| NiFe LDH/THB | 166@0.01A cm^-2^ | 224@0.1A cm^-2^ | 50.4 | – | 7.81 | 3860h@100 mA cm^-2^ | 80% | [60] |
| Ru-S-NiFe LDH | – | 279@0.1A cm^-2^ | 81.65 | 1.19 | 0.093 | 80 h  @1 A cm^-2^ | 95% | [61] |
| NFA-CA (NiFe nanocrystals (core)-NiFe hydroxide (shell) nanostructures) | 169@0.01A cm^-2^ | 236@0.1A cm^-2^ | 49 | – | 6.4 | 1000 h@1 A cm^-2^ | – | [62] |
| (R-COO)_x_σ-Co_3_O_4-x_ | 230@0.01A cm^-2^ | 330@0.1A cm^-2^ | 60.9 | – | 5.86 | 100 h@20 mA cm^-2^ | 90% | [1] |
| CP/Ga_2_O_3_@HEO | 323@0.01A cm^-2^ | 408@0.1A cm^-2^ | 43 | 0.55±  0.07 | 42.82 | 200 cycles | – | [3] |
| FeMoOOH/NF | – | 240@0.1A cm^-2^ | 72.3 | – | 469 | 1000 h@1.5 A cm^-2^ | 100% | [16] |
| FeCoNiMnCr HEA-HEO/CNT | 261@0.01A cm^-2^ | 320@0.5A cm^-2^ | 42.2 | 1.81 | 2.83 | 100 h@100 mA cm^-2^ | 100% | [63] |
| d-(Fe, Ni)OOH | 213@0.01A cm^-2^ | 257@0.1A cm^-2^ | 21.7 | 1.08 | 180 | 17000 cycles | 85% | [64] |
| Ni_2_Fe_1_-O | 359@0.01A cm^-2^ | – | 39 | 4.292 | – | 60 h@10 mA cm^-2^ | – | [49] |
| z-NiFe | – | 178@0.1A cm^-2^ | 28.5 | – | – | 12000 h@1 A cm^-2^ | 100% | [65] |
| Ni_3_S_4_/NiFe-LDH/IF | – | 250@0.22A cm^-2^ | 30.8 | 3.612 | 9.4 | 100 h@500 mA cm^-2^ | 85% | [66] |
| HEOs-Ov (Co, Mn, Fe, Ni, Zn) | 284@0.01A cm^-2^ | – | 53 | – | 16.37 | 200 h@100 mA cm^-2^ | 95% | [67] |
| R-NiFe (S)/NM | – | 310@0.1A cm^-2^ | 41.4 | – | – | ≥80 h@300 mA cm^-2^ | 95% | [68] |
| NiFe-LDH/Ni_4_Mo | 192.5@0.01A cm^-2^ | – | 42 | 7.36 | 6.42 | 150 h@100 mA cm^-2^ | 95% | [69] |

This table compares the OER performance of Ni_2_Fe_0.88_Pr_0.12_-LDH with recently reported state-of-the-art catalysts in alkaline media. Our catalyst exhibits competitive overpotential at both low (0.01 A cm^-2^) and high (500 mA cm^-2^) current densities. Notably, the durability of our catalyst (2190 h to reach a 200 mV overpotential increase at 500 mA cm^-2^) surpasses most reported values, including several that exceed 1000 h, and meets the industrial requirement of ≥800 h for alkaline membrane electrolysis. This exceptional stability is attributed to Pr-induced electronic stabilization, suppressed metal leaching, and the in situ formed robust LDH-substrate interface.
